# Detection of Circulating hcmv-miR-UL112-3p in Patients with Glioblastoma, Rheumatoid Arthritis, Diabetes Mellitus and Healthy Controls

**DOI:** 10.1371/journal.pone.0113740

**Published:** 2014-12-02

**Authors:** Abdul-Aleem Mohammad, Afsar Rahbar, Weng-Onn Lui, Belghis Davoudi, Anca Catrina, Giuseppe Stragliotto, Linda Mellbin, Anders Hamsten, Lars Rydén, Koon-Chu Yaiw, Cecilia Söderberg-Nauclér

**Affiliations:** 1 Experimental Cardiovascular Research Unit, Department of Medicine-Solna, Center for Molecular Medicine, Karolinska Institute, Stockholm, Sweden; 2 Department of Oncology-Pathology, Karolinksa Institutet, Cancer Center Karolinska, Karolinska University Hospital, Stockholm, Sweden; 3 Rheumatology Unit, Department of Medicine, Karolinska University Hospital, Karolinska Institutet, Stockholm, Sweden; 4 Atherosclerosis Research Unit, Center for Molecular Medicine, Karolinska Institute, Stockholm, Sweden; 5 Cardiology Unit, Department of Medicine Solna, Karolinska Institute, Stockholm, Sweden; Saint Louis University, United States of America

## Abstract

**Background:**

microRNAs (miRNA) are 18–22 nucleotides long non-coding RNAs that regulate gene expression at a post-transcriptional level. Human cytomegalovirus (HCMV) encodes at least 26 known mature miRNAs. hcmv-miR-UL112-3p (miR-UL112-3p) is the most well characterized HCMV miRNA, which is suggested to play role in establishment and maintenance of viral latency. Elevated miR-UL112-3p levels have been reported to be present in plasma of patients with hypertension.

**Objectives:**

In this study, we aimed to quantify miR-UL112-3p levels in the plasma/serum of patients with Diabetes Mellitus (DM; from the DIGAMI-2 cohort), Glioblastoma multiforme (GBM), Rheumatoid Arthritis (RA) and Healthy Controls (HC).

**Study Design:**

Total RNA was isolated from plasma/serum samples of 87 patients and controls, a TaqMan miRNA assay was performed to detect miR-UL112-3p and the copy numbers were normalized to 10 ng of total RNA. HCMV IgG and IgM were analysed using ELISA.

**Results:**

HCMV miR-UL112-3p was detected in 14/27 (52%) of DM, 5/20 (25%) of GBM, 1/20 (5%) of RA patients and in 2/20 (10%) of HC, respectively. Anti-HCMV IgG was detected in 85%, 65%, 75% of patients and 70% of HC, respectively. Anti-HCMV IgM was found only in one GBM patient of 87 examined patients and controls.

**Conclusions:**

A higher prevalence of miR-UL112-3p was detected in DM and GBM patients than in RA patients and HC. Elevated levels of miR-UL112-3p and higher prevalence of HCMV IgG were observed in DM patients. Whether the presence of circulating miR-UL112-3p denotes a biomarker of HCMV latency or active replication in patients warrants further investigation.

## Background

The seroprevalence of human cytomegalovirus (HCMV) range from 40–100% worldwide [1]. A primary infection may results in latent and/or persistent infection; during latency, the viral genome is mainly present in myeloid lineage cells and is asymptomatic, but reactivation may cause severe disease in immunocompromised patients [2]. HCMV infection is commonly associated with immune suppression, chronic inflammation and cancer, but the clinical relevance of HCMV in these conditions remains uncertain [3]. Recently, ribosomal profiling of HCMV infected cells revealed translation of over 750 unique HCMV transcripts, which suggest that HCMV is more complex than previously known [4].

MicroRNAs (miRNA) are 18–22 nucleotides long noncoding-RNAs that regulate stability and translation of mRNAs [5]. HCMV encodes at least 26 mature miRNAs (www.mirbase.org) [6]–[8], but the relevance of these in clinical pathologies remains uncertain. hcmv-miR-UL112-3p (miR-UL112-3p) is the most widely studied among HCMV encoded miRNAs and can target both cellular and viral transcripts [9], [10].

In this study, we aimed to quantify miR-UL112-3p levels in the plasma/serum of patients with Diabetes Mellitus (DM), Glioblastoma multiforme (GBM) and Rheumatoid Arthritis (RA) that are suspected to be associated with HCMV infections, and in Healthy Controls (HC) [11]–[16].

## Materials and Methods

### Ethics Statement

For the use of archived plasma or serum samples from patients in different cohorts and healthy blood donors, ethical permissions were approved by the Local Ethical Review Boards of Stockholm (Karolinska Institutet), Borås (Regional ethical board of Göteborg), Uppsala (Academic Hospital), Stavanger (Rogaland Hospital) and Kuopio (Kuopio University Hospital) (Reference numbers for GBM; 2008/628-31/2, RA; 02-251, 2009/1262-31/3, DM; 2008/518-31, 96–164, 97–285, 84–97 and HC; 01-420.). Written informed consent was obtained prior to the enrolment and documented in the study maps at the local clinics. Samples were analysed anonymously after a coding procedure, rendering it impossible for anyone except the clinicians in charge of the patients' care to connect HCMV data to the individual patient.

### Sample Collection

Plasma was obtained from patients diagnosed with DM (n = 27, from the DIGAMI-2 cohort [17]) (10 had myocardial infarction (MI) before the sample was taken), GBM (n = 20), and HC (n = 20). Serum was obtained from 20 RA patients.

### RNA isolation and cDNA synthesis

Total RNA was isolated from 250 µl of plasma/serum using miRVana miRNA isolation kit (Ambion, USA) and quantified using NanoDrop 1000 (Thermo Scientific), cDNA was synthesised using reverse transcription primers from TaqMan miRNA assay and miRNA reverse transcription kit (Applied Biosystems, USA). RNA from *in vitro* HCMV infected human lung fibroblasts and supernatants from uninfected cells were used as positive and negative controls, respectively.

### Cloning and standards preparation

**Figure 1:**
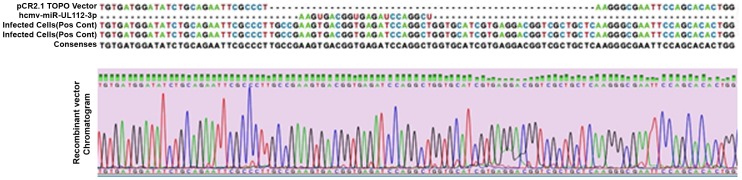
Confirmation of a cloned hcmv-miRUL112-3p TaqMan PCR amplicon from in vitro infected HCMV lung fibroblasts (Positive Control): Alignment of sequences (from above) shows the PCR2.1 TOPO empty plasmid, hcmv-miR-UL112 sequence from miRBase database and last two sequences confirms the PCR product was inserted into the PCR2.1 TOPO plasmid. The lower part represents the chromatogram for the recombinant plasmid.

A miRNA TaqMan real-time PCR reaction was performed with positive control cDNA (HCMV infected lung fibroblasts). The amplicon was electrophoresed on an agarose gel, an approximately 60 bp band was sliced out (54 bp = 27 bp of Stem Loop primer +22 bp miR-UL112-3p+5 bp tail of forward primer) and dissolved in QG buffer (Qiagen, Germany) at 56°C and further purified using Clean all DNA/RNA Clean-up and Concentration Kit (Norgenbiotek, Canada). The purified amplicon was cloned into pCR2.1-TOPO vector and transformed to One shot TOPO10 *E.Coli* competent cells (TOPO TA cloning kit, Invitrogen, USA). Recombinant plasmids were extracted using plasmid mini kit (Qiagen, Germany) and the insert was confirmed by sequencing using M13 primers (Figure 1). Plasmid copy number was determined using the Andrew Staroscik online calculator (www.uri.edu/research/gsc/resources/cndna.html) and concentration was adjusted to 43 ng/µl (Stock) so that the ten time dilutions of stock can cover the entire range of copy numbers and also to determine the limitation of the assay.

### Real-time PCR

**Figure 2:**
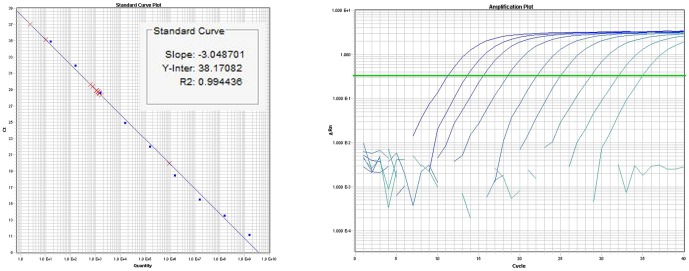
TaqMan assay standard curve plot (left) and amplification plot (right): For miRNA copy number quantification; standard curve was prepared from ten time dilutions of recombinant plasmid containing hcmv-miR-UL112-3p amplicon as insert, which was used as a template in TaqMan miRNA assays.

Triplicate reactions of TaqMan miRNA assays (Applied Biosystems, USA) were performed using TaqMan primers, specific for miR-UL112-3p. Ten-fold diluted recombinant plasmid was used as a template for standard-curve preparation Figure 2. Mean copy number was extrapolated from standard curve and normalised to 10 ng of total RNA.

### Enzyme-Linked Immunosorbent Assay (ELISA)

ELISA was performed using an HCMV IgG kit (Accredited Clinical Virology Laboratory, Karolinska University Hospital, Sweden) [18]–[20] and the Enzygnost Anti-CMV/IgM kit (Siemens, Germany). A detailed procedure for IgG ELISA has been previously described [21]. In brief, for the IgG-ELISA a 96-well plate containing HCMV/control antigen washed three times with washing buffer (0.9%NaCl and 0.05% Tween 20), plasma/serum (1∶500) was added and incubated overnight at 4°C, washed four times and a horse-radish peroxidase conjugated polyclonal rabbit anti-human IgG (1∶5000) was added (Dako, Denmark), incubated for 60 minutes at 37°C. After four washings, reactivity was determined using o-phenylenediamine and the reaction was stopped with 2.5 M sulfuric acid (Merck, Germany). For the IgM-ELISA, non-automated procedure was followed according to the manufacturer. The optical density (OD) was measured at 492 nm and the cut-off value for positivity was OD>0.2.

### Statistical Analysis

Copy numbers of miR-UL112-3p, IgG and IgM OD values were independently evaluated and analysed for significance with Mann-Whitney test using GraphPad Prism. Differences were considered significant at the level of p<0.05.

## Results

**Figure 3:**
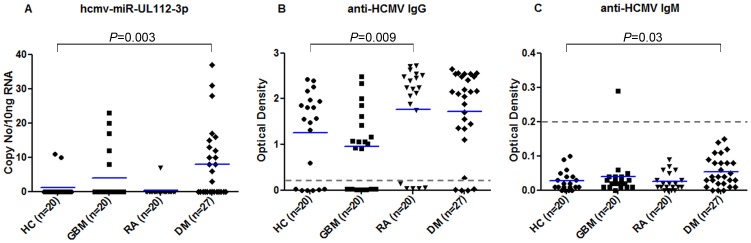
hcmv-miR-UL112-3p TaqMan assay and ELISA: The copy number of hcmv-miR-UL112-3p was extrapolated from the standard curve and normalised to 10 ng of total RNA (A). ELISA for the anti-HCMV IgG (B) and IgM (C) optical density values (OD); the cut-off value for the positivity was OD>0.2 (dotted line in figure 3B and 3C). HC = Healthy Controls, GBM = Glioblastoma multiforme, RA = Rheumatoid Arthritis and DM = Diabetes Mellitus (patients from the DIGAMI-2 cohort).

We examined the prevalence of miR-UL112-3p in 87 plasma/serum samples from HC and patients diagnosed with DM, GBM or RA (Table 1). The mean copy number of miR-UL112-3p was quantified per 10 ng of total RNA. miR-UL112-3p was detected in 52% (14/27, mean copy number 8) of DM patients (4/10 DM had MI), 25% (5/20, mean copy number 4) of GBM patients and in 5% (1/20 mean copy number 1) of RA patients. Among HC 10% (2/20) had 1 mean copy/10 ng RNA. miR-UL112-3p levels were significantly higher in DM patients compared with HCs (*p* = 0.003) Figure 3A.

**Table 1:** Patient characteristics and summarised results.

	Healthy Controls	Glioblastoma multiforme	Rheumatoid Arthritis	Diabetes Mellitus (DIGAMI-2 Cohort)
Serum Samples	20	20		27
**Plasma Samples**			20	
Median age	35	45	57,5	63
**Gender**				
Male	6	9	6	22
Female	14	11	14	5
**Serology**				
IgG Positive	14/20 (70%)	13/20 (65%)	15/20 (75%)	23/27 (85%)
IgM Positive	0/20 (0%)	1/20 (5%)	0/20 (0%)	0/27 (0%)
**hcmv-miR-UL112 TaqMan**				
Positive	2/20 (10%)	5/20 (25%)	1/20 (5%)	14/27 (52%)
Mean copy numbers/10 ng RNA	1	4	0	8

The prevalence of HCMV IgG was 85% (23/27) in DM (10/10 with MI), 65% (13/20) in GBM, 75% (15/20) in RA patients and 70% (14/20) in HC. Significantly higher HCMV IgG OD values were found in RA patients compared with HC (*p* = 0.009), while no difference was observed in DM and GBM patients compared with HC Figure 3B.

**Figure 4:**
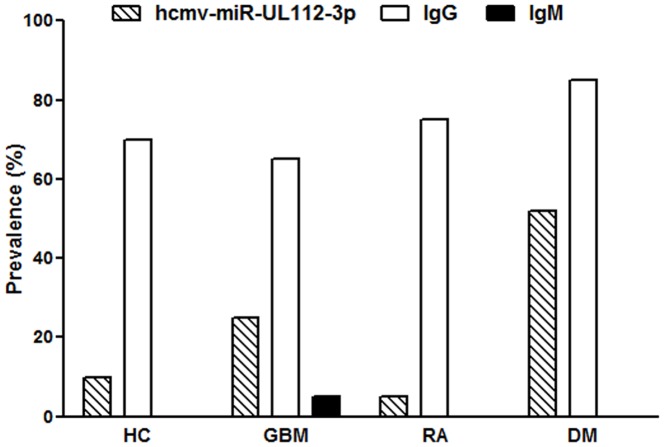
The prevalence of hcmv-miR-UL112-3p was determined using a TaqMan miRNA assay, and the seroprevalence of IgG and IgM against HCMV was detected with ELISA assays in all patients and controls. HC = Healthy Controls, GBM = Glioblastoma multiforme, RA = Rheumatoid Arthritis and DM = Diabetes Mellitus (patients from the DIGAMI-2 cohort).

HCMV IgM was found in one of 20 (5%) GBM patients, all other subjects and controls were IgM negative. Significantly higher HCMV IgM OD values were found in DM patients compared with HC (*p* = 0.03) Figure 3C. The cut-off value for the positivity was OD>0.2 for IgG and IgM ELISAs. No significant correlation was found between HCMV ELISA (IgG and IgM) OD values and miR-UL112-3p levels. The prevalence of miR-UL112-3p, and the seroprevalence of IgG and IgM in all patients and controls are shown in Figure 4.

## Discussion

Inflammation is a driving force for reactivation of latent HCMV [22]. A higher prevalence or activity of HCMV have been reported in RA, DM and GBM patients than in HC [11]–[16]. One previous study indicates that miR-UL112-3p is the only circulating HCMV miRNA highly expressed in hypertensive patients; this HCMV miRNA was associated with increased risk of hypertension [23]. Here, we examined the prevalence of miR-UL112-3p in HC and patients with RA, DM and in GBM. We detected miR-UL112-3p in higher prevalence in DM and GBM patients compared to RA patients and HC.

miR-UL112-3p inhibits translation of the HCMV's IE72 trans-activator, which impacts on active infection and might favour maintenance of latency [10], [24], [25]. miR-UL112-3p also targets cellular transcripts such as MICB and IL-32 and can thereby prevent NK cell recognition and IL-32 mediated TNF-α release, which may also affect the establishment and maintenance of viral latency and persistence [26], [27]. Thus, it is possible that miR-UL112-3p is expressed and mainly exerts its function during latency. The fact that miR-UL112-3p was detected in 52% of the DM patients and 25% of GBM patients, indicates higher virus activity or maintenance in these patients than in RA patients and HCs.

It is not clear from which cells miR-UL112-3p is released into plasma; but monocytes or macrophages, endothelial cells or even tumour cells are plausible sources in conditions related to latent/active HCMV infection in these patients. It is however unclear during which state of virus replication miR-UL112-3p will be released from such cells. Emerging evidence demonstrate that miRNAs are packed into exosomes and microvesicles that are released from cells into the blood circulation [28], [29]; this may occur during latency as well as during active virus replication. We speculate that the presence of miR-UL112-3p in plasma of GBM patients might be a result of released exosomes or microvesicles from the infected tumour cells or inflammatory cells in which this miRNA is expressed. Li et al. also reported the presence of miR-UL112-3p in circulating endothelial cells but not in endothelial progenitor cells in the blood; there was no significant difference between the levels of this miRNA detected in hypertensive patients compared with HC [23]. In the present study, we unfortunately did not have access to patient blood cells to further investigate the presence of miR-UL112-3p in different sub population of the cells.

We conclude that a higher prevalence of miR-UL112-3p was detected in DM and GBM patients as compared to RA patients and HC. Elevated levels of miR-UL112-3p and a higher prevalence of HCMV IgG were observed especially in DM patients, who also had higher IgG OD values than HC. Whether the presence of circulating miR-UL112-3p denotes a biomarker or mediator of disease progression and whether this is linked to active replication or maintenance of HCMV latency in certain patient groups warrants further investigation.
